# The potential role of sucrose transport gene expression in the photosynthetic and yield response of rice cultivars to future CO_2_ concentration

**DOI:** 10.1111/ppl.12973

**Published:** 2019-05-08

**Authors:** Jishuang Zhang, Danfeng Li, Xi Xu, Lewis H. Ziska, Jianguo Zhu, Gang Liu, Chunwu Zhu

**Affiliations:** ^1^ State Key Laboratory of Soil and Sustainable Agriculture, Institute of Soil Science Chinese Academy of Sciences Nanjing 210008 China; ^2^ University of Chinese Academy of Sciences Yuquan Road No. 19A, Beijing 100049 China; ^3^ United States Department of Agriculture, Agricultural Research Service, Adaptive Cropping Systems Lab 10300 Baltimore Avenue, Beltsville MD 20705 USA

## Abstract

The metabolic basis for observed differences in the yield response of rice to projected carbon dioxide concentrations (CO_2_) is unclear. In this study, three rice cultivars, differing in their yield response to elevated CO_2_, were grown under ambient and elevated CO_2_ conditions, using the free‐air CO_2_ enrichment technology. Flag leaves of rice were used to determine (1) if manipulative increases in sink strength decreased the soluble sucrose concentration for the ‘weak’ responders and (2), whether the genetic expression of sucrose transporters OsSUT1 and OsSUT2 was associated with an accumulation of soluble sugars and the maintenance of photosynthetic capacity. For the cultivars that showed a weak response to additional CO_2_, photosynthetic capacity declined under elevated CO_2_ and was associated with an accumulation of soluble sugars. For these cultivars, increasing sink relative to source strength did not increase photosynthesis and no change in *OsSUT1* or *OsSUT2* expression was observed. In contrast, the ‘strong’ response cultivar did not show an increase in soluble sugars or a decline in photosynthesis but demonstrated significant increases in *OsSUT1* and *OsSUT2* expression at elevated CO_2_. Overall, these data suggest that the expression of the sucrose transport genes *OsSUT1* and *OsSUT2* may be associated with the maintenance of photosynthetic capacity of the flag leaf during grain fill; and, potentially, greater yield response of rice as atmospheric CO_2_ increases.

**Abbreviations**
FACE: free‐air CO_2_ enrichment
RT‐PCR: reverse transcription‐PCR
WYJ23: Wuyunjing 23
YD6: Yangdao 6

## Introduction

Based on the continual record of atmospheric CO_2_ measured in Mauna Loa, Hawaii, atmospheric CO_2_ has increased ∼30% (from 315 to 405 µmol mol^−1^) since the mid‐1950s (https://www.esrl.noaa.gov/gmd/ccgg/trends/). Although CO_2_ as a ‘greenhouse gas’ is well recognized, increases in CO_2_ have also been shown in multiple studies to stimulate photosynthesis, growth, fertility and yield of numerous C_3_ crop species, including rice (Baker et al. 1990, Zhu et al. 2012).

However, the degree of stimulation varies depending on the functional level studied. For example, leaf photosynthetic rates can be stimulated by elevated CO_2_, but the extent of photosynthetic stimulation does not necessarily translate into proportional increases in seed yield (Long et al. 2006, Ainsworth et al. 2008, Leakey et al. 2009). This may, in part, be due to a temporal decline in the photosynthetic rate, as the elevated CO_2_ treatment is extended. This is a common phenomenon within C_3_ plants that is referred to as photosynthetic acclimation or downregulation (Chen et al. 2005, Kant et al. 2012). The basis for downregulation may be related to CO_2_‐induced excess photosynthate accumulation in leaves, if sinks for the additional carbon are not available (Stitt 1991, Moore et al. 1999, Haouari et al. 2013, Campany et al. 2017). However, the role of sucrose, the main transporting form of fixed photosynthetic carbon in leaves, is not entirely understood. Specifically, whether the additional photosynthate acquired at elevated CO_2_ is accumulating not only because of sink limitations, but also because of biochemical limits to transport sucrose out of the leaf.

It is generally recognized that there is significant variation to elevated CO_2_ and seed yield stimulation among rice genotypes (Hasegawa et al. 2013, Wang et al. 2016a, b). The link between temporal duration of photosynthetic stimulation with elevated CO_2_ (i.e. lack of downregulation) and the observed stimulation of seed yield is therefore a matter of interest in selecting for greater seed yield responsiveness to rising atmospheric CO_2_ among rice lines.

In situ assessments of rice to elevated CO_2_ using free‐air CO_2_ enrichment (FACE) have demonstrated that genotypes with greater yield response to CO_2_ also had bigger panicles and additional spikelets relative to genotypes with a smaller yield response, suggesting bigger sink capacity (Hasegawa et al. 2013, Zhu et al. 2015). This indicated a potential increase in the sink:source ratio, and an enhanced capacity to accommodate additional photosynthate and avoid downregulation during grain development under elevated CO_2_ conditions (Zhu et al. 2014). However, the role of sucrose transport per se under elevated CO_2_ was not examined.

To determine if a mechanistic link between leaf photosynthetic acclimation and sucrose transportation exists among rice lines differing in yield stimulation to elevated CO_2_, two ‘weak’ cultivars, Wuyunjing 23 (WYJ23) and Nanjing 9108 (NG9108; both ∼10% increase in seed yield at elevated CO_2_) were compared to a ‘strong’ cultivar [Yangdao 6, (YD6); ∼30% increase in seed yield at elevated CO_2_; Zhu et al. 2014]. Our objectives were to determine: (1) if an increase in the sink to source ratio (by removal of source leaves 2 and 3 below the flag leaf) mitigated photosynthetic downregulation in the ‘weak’ cultivars and (2), whether the occurrence of photosynthetic downregulation to elevated CO_2_ was associated with changes in the expression of genes associated with sucrose transportation.

## Materials and methods

### Experimental site description

The study was conducted at the FACE platform located in Zongcun village (32°35′5″N, 119°42′0″E), Yangzhou city, Jiangsu province in Eastern China. This location represents a typical rice‐wheat rotation system within a subtropical marine climatic zone (Zhu et al. 2012). The soil is classified as a Shajiang‐Aquic Cambiosol with a sandy loam texture. Operational details for the FACE system at this location have been described previously (Okada et al. 2001). It consists of three identical 17‐m‐diameter octagonal rings with the CO_2_ at the center of each ring ∼200 µmol mol^−1^ higher than at ambient conditions (representing elevated CO_2_ conditions) and three comparison rings without supplemental CO_2_ (representing ambient CO_2_ conditions). During the seasons in 2014 and 2015, the average daytime CO_2_ values were 394 and 590 µmol mol^−1^ and 395 and 588 µmol mol^−1^ for the ambient and elevated FACE rings, respectively. The average air temperature from planting to harvest was 22.1 and 24.8°C for 2014 and 2015, respectively.

### Rice cultivation and sample pre‐treatment

Based on their relative yield responses to enhanced CO_2_, three rice cultivars, WYJ23, Nanjing 9108 (NG9108) (Japonica) and YD6 (Indica), were selected. Selection was based on their differential yield responses to elevated CO_2_, with WYJ23 and NG9108 demonstrating weaker stimulation relative to YD6 (ca 10 vs. 30%, respectively, Table 1; Zhu et al. 2015). Seeds of each variety were sown at ambient CO_2_ in late May, 2014 and 2015, and seedlings were manually transplanted to ambient and elevated rings on June 21 and June 17 for 2014 and 2015, respectively. Two seedlings per hill with 24 hills per m^2^ were the planting density for all six rings. Phosphorous (P) and potassium (K) were applied as compound fertilizers at 9 g P_2_O_5_ m^−2^ and 9 g K_2_O m^−2^, using a basal dressing 1 day before transplanting. Nitrogen (N, at 22.5 g N m^−2^ each season) was applied as a basal dressing (40% of the seasonal total), 1 day prior to transplanting and as a top dressing at early tillering (30% of the seasonal total) and again at the panicle initiation stage (30% of the seasonal total).

**Table 1:** Effects of FACE on three rice cultivars, WYJ23, NG9108 and YD6 over two growth seasons (2014 and 2015). % Change is relative difference at elevated to ambient CO_2_. Values are means of three replicates. Values and statistics are from Zhu et al. 2015. ns, not significant.

Data from 2014 growing season and the data of YD6 combined from Zhu et al. (2015)						
Variety	CO_2_	Panicle number (m^−2^)	Spikelets per panicle	Filled pikelet ratio	Weight per grain	Yield (g m^−2^)
WYJ23	% Change	14.5	−7.1	3.4	1.8	12.4 ns
YD6	% Change	11.2	6.8	5.9	2.3	29.6*
Data from 2015 growing season						
Variety	CO_2_	Panicle number (m^−2^)	Spikelets per panicle	Filled pikelet ratio	Weight per grain	Yield
WYJ23	% Change	13.0	−6.0	2.9	0.0	11.0 ns
NG9108	% Change	12.3	−5.4	1.2	1.4	9.1 ns
YD6	% Change	10.6	11.5	2.6	3.1	29.5*

At the heading stage in each CO_2_ treatment, two tillers of WYJ23 and NG9108 (i.e. the weaker CO_2_ response cultivars), were chosen and tagged in all replicates, and the 2nd and 3rd leaves were removed from one of the tillers to increase the sink:source ratio in 2014 and 2015. It has been shown that genotypes with greater response to CO_2_ have an adequate sink capacity (Hasegawa et al. 2013, Zhu et al. 2015). Therefore, leaves were not removed for YD6, and comparisons were made between flag leaves of the different cultivars.

### Photosynthesis gas exchange measurements

Measurements of leaf net photosynthesis were conducted in situ during the grain filling stage for each cultivar, using a portable photosynthesis system equipped with blue and red LED light sources (LI‐6400; LI‐COR, Lincoln). Photosynthesis measurements began at grain fill and continued for a 2‐day period: September 19–21 for WYJ23 and YD6 in 2014, September 13, 14 for NG9108 and September 17, 18 for WYJ23 and September 8, 9 for YD6 in 2015. Measurements were made at a saturating photosynthetic photon flux density of 1800 µmol m^−2^ s^−1^. Leaf temperature was set to 30°C and air flow rate was set to 500 µmol s^−1^.

### Sampling and biochemical analyses

Following determination of leaf photosynthesis, during the first 2 days of grain filling, two of the measured flag leaves from all cultivars and experimental treatments were sampled from 9:30–14:30 (Beijing time). Both leaves were stored in liquid nitrogen until analysis. Chosen tillers were divided into panicle and flag leaf for dry weight (at 80°C for 72 h), and then flag leaves were ground to determine soluble sugar and nitrogen content as published in Olano et al. (2006).

An anthrone colorimetric method was used to measure the concentration of soluble sugars (Buysse and Merckx 1993). Leaf tissue nitrogen concentration was measured using an elemental (Carbon‐Hydrogen‐Nitrogen) analyzer (PE2400 series II CHNS/O).

The flag leaves (stored in liquid N) were used to quantify sucrose transport genetics using an established procedure (Lin 2010, Wang et al. 2016a, b): 1 µg of total RNA treated with DNase I (TaKaRa) was used for reverse transcription‐PCR (RT‐PCR). RT was performed using PrimeScript TM RT Master Mix (TaKaRa). PCR was performed at 37°C for 15 min, 85°C for 5 s and cDNA was stored at 4°C. Quantitative RT‐PCR was carried out on a CFX96 real‐time PCR system (Bio‐Rad Laboratories, Hercules) using the SYBR Premix Ex Taq TM (TaKaRa) with 35 cycles of 95°C for 5 s and 60°C for 30 s. Gene expression data analysis included normalizing of *OsSUT1* and *OsSUT2* Ct values to the housekeeping gene *Rac 1* (X16280.1). The expression levels of *OsSUT1* and *OsSUT2* were calculated as *E*‐ΔΔCt (analysis in sequence; *OsSUT1*: F‐5′ CTGTGATTTTCCTGTCCCTG 3′ and R‐5′ AACACTGCTAGTGGACCAGT 3′, *OsSUT2*: F‐5′ AGGAGGAGAGGTCACCGATAA 3′ and R‐5′ CCAACATCCAATGTACAACAGCA 3′) and the primer sequences mentioned before were used in this PCR study. Quantitative expression of these genes was used to represent sucrose transport capacity in the current study. Housekeeping gene primer sequences were: Rac 1: F‐5′ GTACCCGCATCAGGCATCT 3′ and R‐5′ TCCATCTTGGCATCTCTCAG 3′.

### Statistical analysis

Data were analyzed using the SPSS statistical software (SPSS 19.0; SPSS Inc.) and Excel 2016 for Windows 10. The CO_2_ treatments (ambient and elevated) were analyzed as a randomized complete block, and the sink:source manipulation (removal of leaves) was analyzed as a split‐plot treatment. Each treatment group consisted of three replicates. Analysis of variance (anova) was used to test for significant treatment differences.

## Results

### Yield components and single‐panicle weight

YD6 showed a consistently greater yield response than WYJ23 and NG9108 at elevated CO_2_ (Table 1). Among the yield components examined, the effect of elevated CO_2_ was positive for spikelets per panicle in YD6, while negative in WYJ23 and NG9108. Leaf removal and enhanced sink:source ratio consistently, but not significantly (i.e. *P* was 0.095 and 0.068 for WYJ23 and NG9108, respectively) lowered single‐panicle dry weight in response to elevated atmospheric CO_2_ (Table 2).

**Table 2:** Effects of sink:source treatment on single‐panicle dry weight for WYJ23 and NG9108 under elevated CO_2_ in 2014 and 2015. ‘Enhanced’ indicates the increased sink:source ratio through leaf removal, and the unaltered sink:source ratio is represented by ‘Control’. Values are mean of three replicates. *P* > 0.1; †*P* ≤ 0.1; *P ≤ 0.05; **P ≤ 0.001.

Variety	Sink:source	Single‐panicle dry weight (g)	
WYJ23 (2014)	Control	1.314†	
	Enhanced	1.176	
WYJ23 (2015)	Control	2.231†	
	Enhanced	2.068	
NG9108 (2015)	Control	2.733†	
	Enhanced	2.573	
anova result	WYJ23 (2014)	WYJ23 (2015)	NG9108 (2015)
*P*‐Value	0.088	0.095	0.068

### Leaf net photosynthesis and photochemistry

**Figure 1:**
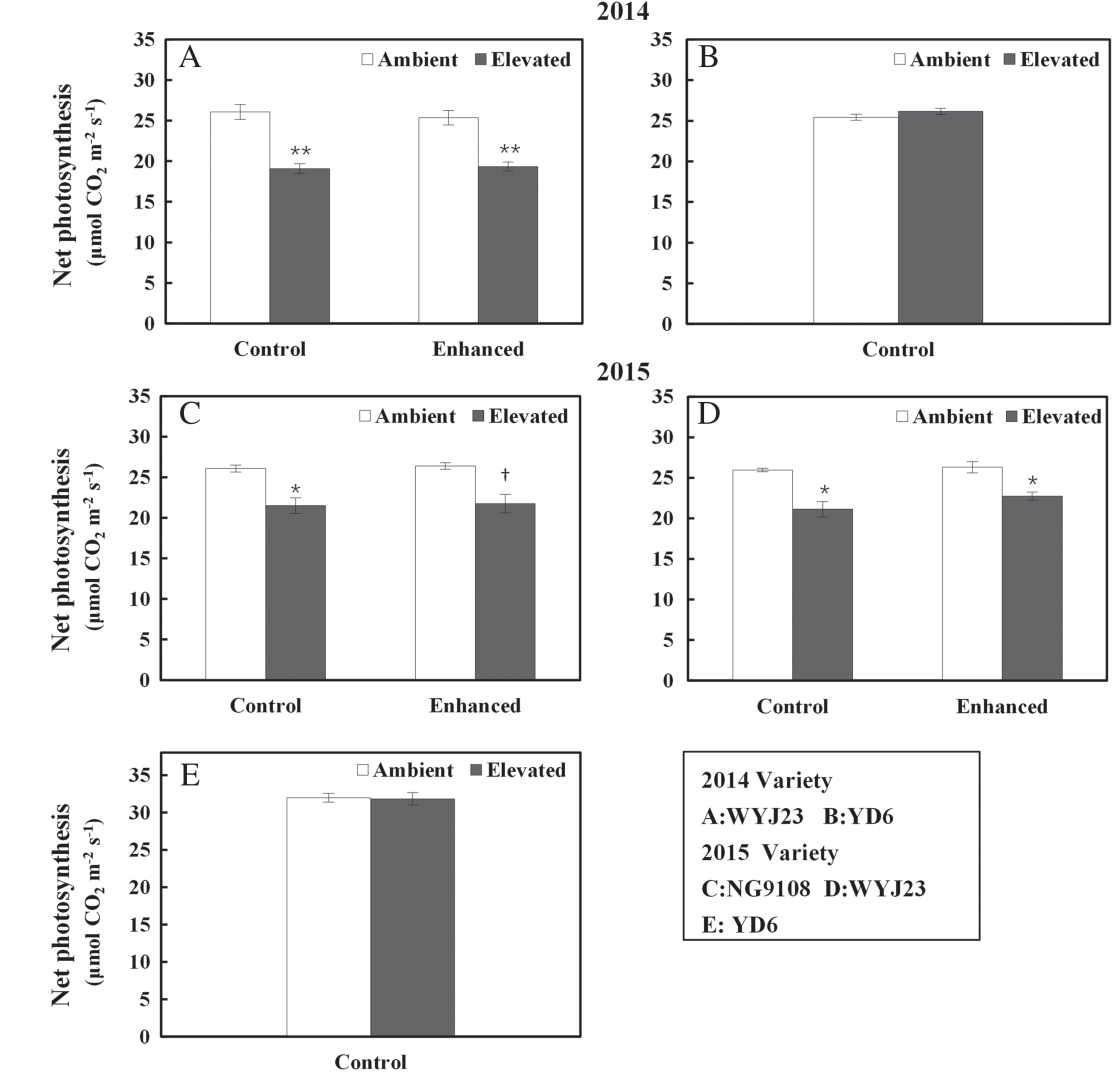
Net photosynthesis rate (µmol CO_2_ m^−2^ s^−1^) of flag leaves for three rice cultivars, WYJ23, NG9108 and YD6 grown at ambient and elevated CO_2_ in each sink:source treatment. Measurements were made at the same CO_2_ condition (590 µmol mol^−1^). ‘Enhanced’ indicates the increased sink:source ratio through leaves removal and the unaltered sink:source ratio is represented by ‘Control’. Bars represent average values of three replicates with standard errors. Symbols indicate significant differences in sink:source treatment for each cultivar as a function of CO_2_ treatment. ns, not significant. *P* > 0.1; †*P* ≤ 0.1; *P ≤ 0.05; **P ≤ 0.001.

At elevated CO_2_ conditions, relative to ambient CO_2_, significant photosynthetic downregulation was observed for WYJ23 and NG9108 (Fig. 1A, C, D). Increasing sink:source ratios through leaf removal did not negate photosynthetic downregulation for these cultivars. In contrast, for YD6, net photosynthetic rate showed no downregulation in response to elevated CO_2_ (Fig. 1B, E).

Consistent with downregulation, a decrease in the photosynthetic rate of the flag leaf was associated with a significant decline in leaf N concentration at elevated CO_2_ conditions (Table 3). This decline was observed for WYJ23 and NG9108 and was not altered by sink:source manipulation (Table 3). In contrast, YD6 did not show any significant change in leaf N concentration (Table 3).

**Table 3:** Nitrogen content of flag leaves for the rice cultivars, WYJ23, NG9108 and YD6 in each treatment. Values are the average of three replicates of each treatment. ‘Enhanced’ indicates the increased sink:source ratio through leaves removal, and the unaltered sink:source ratio is represented by ‘Control’. Two‐way anova for CO_2_ and sink:source treatment is used in WYJ23 and NG9108 and one‐way anova for CO_2_ treatment is used in YD6. E/A, Elevated/Ambient; ‐, no data; ns, not significant. *P* > 0.1; †*P* ≤ 0.1; *P ≤ 0.05; **P ≤ 0.001.

Year	Variety	CO_2_	Sink:source	N (%)
2014	WYJ23	Ambient	Control	2.39
		Elevated	Control	2.09
		Changes (E/A)		−12.7
		Ambient	Enhanced	2.47
		Elevated	Enhanced	2.28
		Changes (E/A)		−7.7
	YD6	Ambient	Control	2.56
		Elevated	Control	2.41
		Changes (E/A)		−5.9
2015	WYJ23	Ambient	Control	2.24
		Elevated	Control	1.96
		Changes (E/A)		−12.7
		Ambient	Enhanced	2.18
		Elevated	Enhanced	1.94
		Changes (E/A)		−11.0
	NG9108	Ambient	Control	2.19
		Elevated	Control	1.83
		Changes (E/A)		−16.6
		Ambient	Enhanced	2.19
		Elevated	Enhanced	1.86
		Changes (E/A)	Control	−15.1
	YD6	Ambient	Control	2.35
		Elevated	Control	2.14
		Changes (E/A)		−8.6
anova result		N (%)		
		WYJ23	NG9108	YD6
2014	CO_2_	*	‐	ns
	Sink:source	ns	‐	‐
	CO_2_ × sink:source	ns	‐	‐
2015	CO_2_	*	*	ns
	Sink:source	ns	ns	‐
	CO_2_ × sink:source	ns	ns	‐

### Soluble sugars accumulation and *OsSUTs* expression

**Figure 2:**
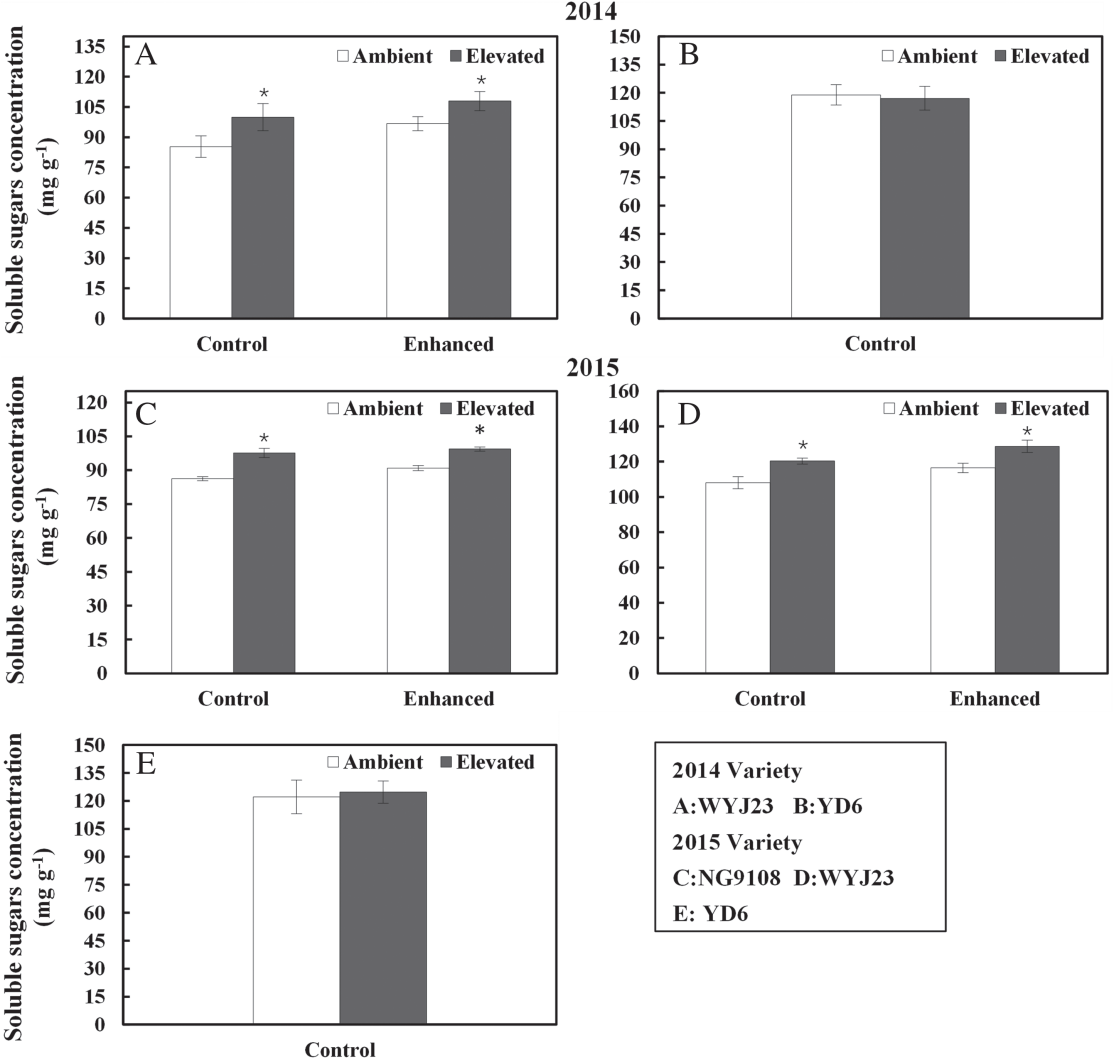
Soluble sugars concentration in flag leaves for three rice cultivars, WYJ23, NG9108 and YD6 grown at ambient and elevated CO_2_ in combination with sink:source treatments. ‘Enhanced’ indicates the increased sink:source ratio through leaves removal, and the unaltered sink:source ratio is represented by ‘Control’. Bars represent average values of three replicates with standard errors. Symbols indicate the significant difference in a given sink:source treatment for each cultivar as a function of CO_2_ treatment. ns, not significant. *P* > 0.1; †*P* ≤ 0.1; *P ≤ 0.05; **P ≤ 0.001.

At elevated CO_2_ conditions, a significant increase in leaf soluble sugar concentrations for WYJ23 and NG9108 with and without removal of additional source leaves was measured (Fig. 2). In contrast, no change in soluble sugar concentration in the flag leaf was observed for YD6. No significant differences in leaf soluble sugar concentration were observed for WYJ123 or NG9108 as a function of CO_2_ concentration (Fig. 2A, C, D).

**Figure 3:**
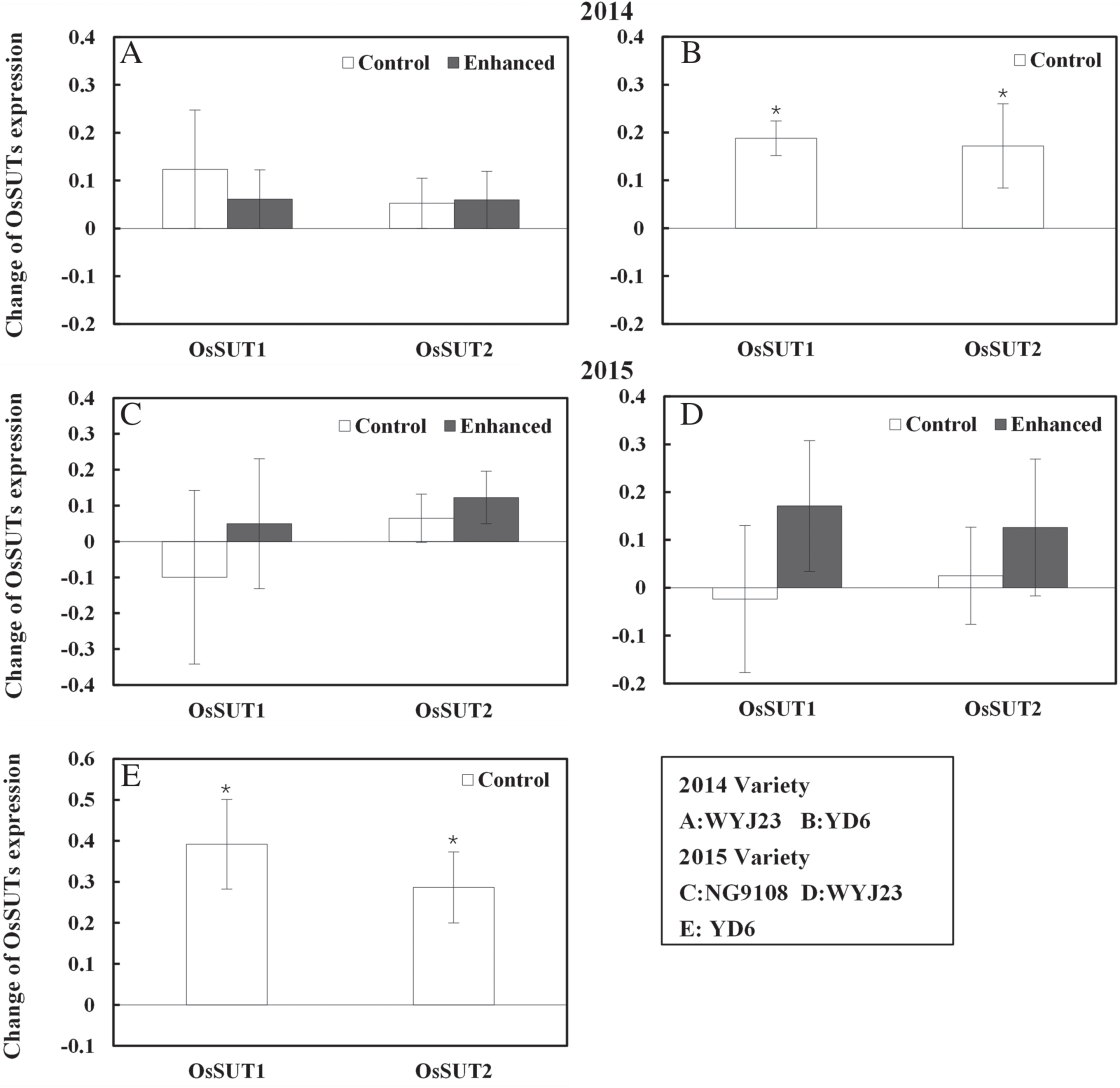
Change of *OsSUT1* and *OsSUT2* (*OsSUTs*) expression level of flag leaves under elevated CO_2_ for three rice cultivars in each sink:source treatment. ‘Enhanced’ indicates the increased sink:source ratio through leaves removal, and the unaltered sink:source ratio is represented by ‘Control’. Bars represent the average (*E*‐A)/A (relative change at elevated CO_2_ to those at ambient CO_2_) of three replicates for *OsSUTs* expression level with relative standard errors. Symbols indicate the significant difference for the gene expression as a function of CO_2_ treatment. ns, not significant. *P* > 0.1; †*P* ≤ 0.1; *P ≤ 0.05; **P ≤ 0.001.

*OsSUT1* and *OsSUT2* represent the sucrose transport genes for rice and are characterized as necessary for sucrose export from source leaves. For YD6, *OsSUT1* and *OsSUT2* (*OsSUTs*) expression increased significantly in response to elevated CO_2_. In contrast, the enhanced source treatment or elevated CO_2_ had no effect on *OsSUTs* expression for WYJ23 and NG9108 (Fig. 3).

**Figure 4:**
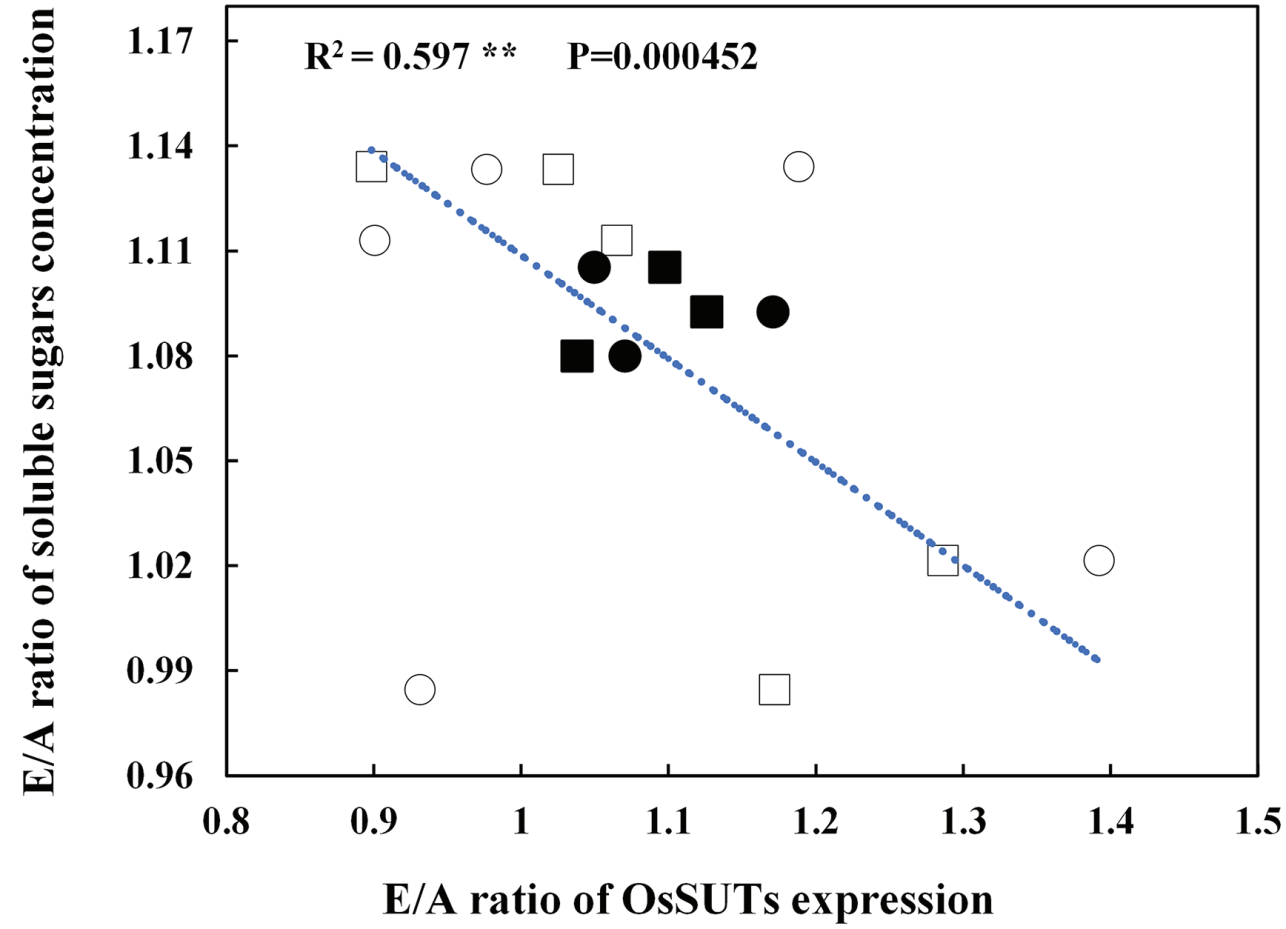
Relationship between E/A (relative values at elevated CO_2_ to those at ambient CO_2_) ratio of OsSUTs expression level and E/A ratio of soluble sugars concentration in flag leaves. Values are the average of three replicates. Circles represent the values of OsSUT1 expression level, and boxes represent the values of *OsSUT2* expression level. The open symbols indicate the values of control sink:source treatment, and the solid symbols indicate the values of enhanced sink:source treatment. R^2^ = 0.591, *P*‐value = 0.000452, **P ≤ 0.001.

The elevated/ambient CO_2_ ratios of soluble sugars and *OsSUT* expression were analyzed for all cultivars, treatments and years. The soluble sugar ratio was negatively correlated with the *OsSUTs* expression ratio (Fig. 4, *P* < 0.001). This suggested that if *OsSUT* expression was insufficient, soluble sugars would accumulate under elevated CO_2_.

## Discussion

When grown at projected, elevated levels of CO_2_, there is consistent intraspecific variation among crop cultivars in growth and yield, some showing a strong stimulation of yield, others little or no stimulation (Ziska et al. 2014, Bishop et al. 2015) Understanding the basis for this variation may be essential in identifying those cultivars that can convert additional CO_2_ into greater seed yield.

While the basis for intraspecific variation is likely to be multifactorial, photosynthetic capacity over time is of obvious importance. Under elevated CO_2_ conditions, inadequate sinks for additional carbon may result in a surplus accumulation of photosynthate at the leaf level, with eventual downregulation of photosynthesis (Lin et al. 1997, Shimono and Okada 2013, Ziska et al. 2014, Burnett et al. 2016, Ruiz‐Vera et al. 2017). This had been reported for numerous C_3_ crop species including rice (Ono et al. 2003, Zhu et al. 2014).

At present, the role of sucrose transport in feedback inhibition of photosynthesis is unclear. Sucrose transport, an essential part in the carbohydrate distribution process, can be sensitive to environmental changes, e.g. cold or heat, with consequences for photoassimilate distribution and photosynthetic downregulation (Takahashi et al. 2017, Zhou et al. 2017). However, it is uncertain whether the capacity of sucrose export from source leaves is related to the overall photosynthetic response to elevated CO_2_.

Is the extent of downregulation and/or expression of sucrose transport related to a relative yield stimulation among rice cultivars in response to additional CO_2_? In this study, YD6 had a higher (∼twofold) yield response relative to cultivars WYJ23 and NG9108 at elevated CO_2_ under field conditions. The yield responses are, in general, in agreement with the observed changes in the source:sink and photosynthetic downregulation for the cultivars WYJ23 and NG9108.

It is interesting to note that when sink limitation was diminished by increasing the ratio of carbon sinks to source for these two cultivars, photosynthetic downregulation was still observed (Table 2, Fig. 1). This suggested that eliminating sink limitation per se did not mitigate photosynthetic downregulation under elevated CO_2_ conditions. Rather, it suggested that additional factors could be involved, including sucrose transport capacity. For example, sucrose transporter genes *OsSUT1* and *OsSUT2* have been reported to play an essential role in the sucrose apoplastic loading into the phloem (Aoki et al. 2003, Eom et al. 2011, Braun et al. 2014, Chen et al. 2015).

At elevated CO_2_ conditions, the enhancement in gene expression of *OsSUT1* and *OsSUT2* was negatively correlated with soluble sugar accumulation (Fig. 4), consistent with previous research on chilling temperatures (Takahashi et al. 2017). In the current experiment, the relative variation in gene expression among the three lines, relative to yield stimulation, is of interest in the context of CO_2_. In YD6 e.g. additional photosynthate did not accumulate in the source leaves and photosynthetic downregulation was not observed at elevated CO_2_ conditions. Conversely, even without the sink restriction, gene expression of *OsSUT1* and *OsSUT2* was not upregulated significantly under elevated CO_2_. Overall, the change in gene expression was inversely proportional to the accumulation of photosynthates at elevated CO_2_ among the examined cultivars.

For this study, the decrease in leaf‐nitrogen concentration of WYJ23 and NG9108 (japonica) was greater than YD6 (indica) at elevated relative to ambient CO_2_. There are other noted differences between japonica and indica in regard to stomatal conductance, root size and nitrogen distribution (Kant et al. 2012, Shimoda and Maruyama 2014, Muryono et al. 2017). It is possible that insufficient sucrose transport under elevated CO_2_ may be associated with the relative N shortage. For example, N deficiency could alter the distribution of sucrose across plant organs (Lemoine et al. 2013). In addition, sugar accumulation in functional leaves can inhibit SUT expression and activity (Chiou and Bush 1998, Cordoba et al. 2015). However, additional indica and japonica comparisons would be necessary to validate the role of nitrogen in sucrose gene expression at elevated CO_2_.

Overall, the relative stimulation of yield at elevated CO_2_ was correlated with a lack of photosynthetic downregulation that in turn reflected higher expression levels of *OsSUT1* and *OsSUT2* in this study. While a wider array of rice cultivars needs to be examined to confirm these results, these initial data indicate that stimulation of sucrose transport genes during grain filling could be associated with greater yield sensitivity to rising CO_2_. Given the global importance of rice in the context of future food security, any mechanism that can enhance the conversion of additional CO_2_ into seed yield would be of interest in that regard.

## Author contributions

J.S.Z., D.F.L. and X.X. performed the experiments and drafted the manuscript. C.W.Z. conceived the study. C.W.Z., L.H.Z, J.G.Z. and G.L. participated in its design. J.S.Z., D.F.L., C.W.Z. and L.H.Z. edited the manuscript. All authors declare that they have no conflict of interest.
